# A novel Human Conception Optimizer for solving optimization problems

**DOI:** 10.1038/s41598-022-25031-6

**Published:** 2022-12-14

**Authors:** Debasis Acharya, Dushmanta Kumar Das

**Affiliations:** Department of Electrical and Electronics Engineering, National Institute of Technology Nagaland, Dimapur, 797103 India

**Keywords:** Engineering, Biomedical engineering, Electrical and electronic engineering, Computational science, Computer science

## Abstract

Computational techniques are widely used to solve complex optimization problems in different fields such as engineering, finance, biology, and so on. In this paper, the Human Conception Optimizer (HCO) is proposed as a novel metaheuristic algorithm to solve any optimization problems. The idea of this algorithm is based on some biological principles of the human conception process, such as the selective nature of cervical gel in the female reproductive system to allow only healthy sperm cells into the cervix, the guidance nature of mucus gel to help sperm track a genital tracking path towards the egg in the Fallopian tube, the asymmetric nature of flagellar movement which allows sperm cells to move in the reproductive system, the sperm hyperactivation process to make them able to fertilize an egg. Thus, the strategies pursued by the sperm in searching for the egg in the Fallopian tube are modeled mathematically. The best sperm which will meet the position of the egg will be the solution of the algorithm. The performance of the proposed HCO algorithm is examined with a set of basic benchmark test functions called IEEE CEC-2005 and IEEE CEC-2020. A comparative study is also performed between the HCO algorithm and other available algorithms. The significance of the results is verified with statistical test methods. To validate the proposed HCO algorithm, two real-world engineering optimization problems are examined. For this purpose, a complex 14 over-current relay based IEEE 8 bus distribution system is considered. With the proposed algorithm, an improvement of 50% to 60% in total relay operating times is observed comparing with some existing results for the same system. Another engineering problem of designing an optimal proportional integral derivative (PID) controller for a blower driven patient hose mechanical ventilator (MV) is examined. A significant improvement in terms of response time, settling time is observed in the MV system by comparing with existing results.

## Introduction

The optimization method is a numerical computational method to find the optimal solution of a real-time problem in a diversified field such as engineering, management, finance and so on^1–6^. Analytical optimization methods are complex and time-consuming processes to get an optimal solution of a complex optimization problem. Again, heuristic optimization methods are problem-dependent techniques^7^. They need particularities of an optimization problem. They are too greedy to get trapped in a local solution. Meta-heuristic methods are problem independent. They can provide an acceptable solution without guaranteeing optimality^8^. A simple concept can be implemented easily to make a metaheuristic algorithm to solve a complex problem quickly. Such algorithms can be applied in any optimization problem without altering the structure of the algorithm. In comparison to analytical based optimization algorithms, a metaheuristic algorithm is free from derivation action to find optimal solution. Thus, a real time problem can be solved by any metaheuristic algorithm where it needs only the information of input and output of the system^9^. Therefore, researchers are giving priority to develop metaheuristic algorithms using natural concepts such as the concept of evolution, the behaviour of natural creatures and hunting procedure followed by animals, and so on^9–11^.

In metaheuristic algorithms, they start with exploring new solutions and transmitting them to exploit the best solution for a given problem^11^. In the exploitation phase of the metaheuristic algorithm, a new solution is produced based on the best solution available in the population. Thus, metaheuristic algorithms use an exploration and exploitation process to avoid local trapping problems and converge towards the optimal solution. Moreover, by striking a proper balance in the exploration and exploitation phases of such an algorithm, the local optimality problem of traditional methods can be avoided^12,13^.

In this paper, the Human Conception Optimizer (HCO) is proposed as a novel metaheuristic algorithm to solve any optimization problems. The HCO algorithm is inspired by the biological principle of natural human conception. For successful natural conception by a female, the fittest sperm of a male must fertilise a mature egg. The movement of sperm in the genital tracking path towards the egg is a unique characteristic. Sperm also use a unique technique to avoid environmental obstacles in the female reproductive system^14^. In Ref.^15^, the authors have proposed a sperm motility algorithm (SMA) using the principle of chemoattractant secreted by the ovum to guide sperm movement in the female reproductive system. In Ref.^16^, the authors have proposed a Sperm Swarm Optimization (SSO) algorithm based on the temperature guidance for sperm to search for the egg in the female reproductive system. In Ref.^17^, the authors have proposed a multi-objective sperm fertilization procedure (MOSFP) as a modified form of SSO for multiobjective optimization problems. The general principles of natural conception have been used to develop novel algorithms^15–17^. The conception process from the point of view of sperm movement towards the egg in the female reproductive system is modeled in this paper. The concept of the natural selection of healthy sperm allowed by cervical gel to enter into the cervix, their unsymmetrical trajectories during movement, sperm guidance mechanism, flaggers’ moving characteristics with very spatial hyperactivation principle of sperm during the fertilisation of a mature egg are utilised as the ideas of the proposed Human Conception Optimizer (HCO) algorithm. Such concepts are utilized for the first time to develop a metaheuristic algorithm. The efficiency of the proposed algorithm is validated with standard IEEE CEC-2005 and CEC-2020 benchmark functions. A comparative study is also performed between the HCO algorithm and some existing algorithms for the benchmark functions. The statistical significance of test results is also studied with two types of non-parametric tests, such as the Friedman test and the Wilcoxon signed rank test. The applicability of the HCO algorithm in engineering problems has been validated for two different cases. In the first case, the optimal coordination of over-current relays in a power distribution network is studied for an IEEE 8-bus system. In the second case, an optimal PID controller is designed for the human respiratory ventilation system.

## Related works

In the literature, different analytical solutions are found, such as Quadratic Programming (QP), Dynamic Programming (DP), Lagrangian method for optimization problems. All such methods are based on deferential operators. They start searching for an optimal solution nearest to the initial point. Insufficient gradient data may lead them to a local solution. Thus, a limited application of such methods is found for real-world, complex optimization problems. In this regard, metaheurestic methods are found to be better than analytical methods in the literature^1–3^. There are mainly three types of metaheuristic algorithm found in literature such as: physics based, swarm intelligence based and evolutionary metaheuristic algorithm. In evolutionary metaheuristic algorithms, the laws of natural evolution are used^12^. The search technique begins with a random generation population where the best solutions are combined and mutated to form new solutions. Genetic algorithm (GA)^18^ is one of evolutionary algorithms based on the Darwinian evolution concept. Other evolutionary algorithms are such as Evolution Strategy (ES)^19^, Genetic Programming (GP)^20^ etc. Some metaheuristic algorithms are inspired by well-known physical laws of the nature. Some of them are Simulated Annealing (SA)^21^, Gravitational Search Algorithm (GSA)^22^, Big-Bang Big-Crunch (BBBC)^23^, Atomic orbital search (AOS)^24^, Charged System Search (CSS)^25^ etc. Some swarm-based metaheuristic algorithms are inspired by the social behaviour of animals such as Particle Swarm Optimization (PSO)^6^, Ant Colony Optimization (ACT)^26^ etc. A swarm intelligence based algorithm employs a large number of particles to cover a search space, and the optimum answer is discovered by following the best location along their pathways^27^. Particles with their best solutions and the best one obtained so far in the swarm are used to update the particle position. Many other swarm based algorithms are found in literature such as Whale Optimization Algorithm (WOA)^28^, Grey Wolf Optimization (GWO) algorithm^5^, Sailfish Optimizer (SFO)^29^, Bottlenose Dolphin Optimizer^30^. Some human behavior-based metaheuristic methods are also found in literature such as Teaching Learning Based Optimization (TLBO)^31^, Group Search Optimizer (GSO)^32^, Imperialist Competitive Algorithm (ICA)^33^, Class Topper Optimization (CTO)^4^, Criminal search optimization^34^ and so on.

Generally, a metaheuristic algorithm starts with a random initialization of the search variables within specified range. The convergence performance of such an algorithm depends on the correct selection of the initial value or position of the searching agent. An improper selection of initial value, which may be in a different direction where the actual solution may exist, can lead towards the wrong solution. Thus, the selection of the initial position of the search variable has an impact on the convergence performance of a metaheuristic algorithm^12,13^. Another issue in metaheuristic algorithms is the trapping problem in local solutions during the exploration and exploitation stages of such algorithms. Thus, the improper selection of initial value or position of search agents and local stack problems of such metaheuristic methods need to be solved to get an efficient optimizer. In Ref.^35^, authors presented a theory named the No Free Lunch (NFL) theory and proved that there is a universal best optimization method as all such methods perform similarly for all possible optimization-based problems. Therefore, many authors are involved in developing specific problem-based optimizers with the aim of getting global and local search strategies. In this regard, an attempt has been made to solve such issues by developing the Human conception optimizer (HCO). The unique features of the human conception process justify the development of such an algorithm. The HCO algorithm can solve the issues as stated above by resembling some spatial techniques of the conception process, which are discussed in the next section. A list of some existing optimization methods has been presented in Table 1.

**Table 1:** Different existing nature inspired optimizer.

Algorithm	Inspiration source	Year
Genetic algorithm (GA)^18^	Genetic valuation	1992
Particle swarm optimization (PSO)^6^	Social behavior of birds	1995
Genetic algorithms^18^	Concept of genetic evaluation	1992
Harmony Search (HS)^36^	Music player	2001
Genetic Programming (GP)^20^	Genetic concept	2005
Ant colony optimization (ACO)^26^	Ant colony	2006
Cat Swarm Optimization (CSO)^37^	Cat behavior	2006
Monkey Search^38^	Monkey	2007
Bee Collecting Pollen Algorithm (BCPA)^39^	Bees	2008
Cuckoo Search (CS)	Cuckoo	2009
Dolphin Partner Optimization (DPO)^40^	Dolphin	2009
Group Search Optimizer (GSO)^32^	Animal searching	2009
Gravitational search algorithm (GSA)^22^	Gravitational attraction of heavy mass	2009
Fireworks Algorithm (FA)^41^	Fireworks explosion	2010
Charged System Search (CSS)^25^	The Coulomb and the Newtonian law	2010
Teaching Learning Based Optimization (TLBO)^31^	Student-teacher interaction	2011
Krill Herd (KH)^42^	Krill herd	2012
Flower Pollination Algorithm^43^	Flower Pollination	2012
Water Cycle Algorithm (WCA)^44^	Water cycle process	2012
Mine Blast Algorithm (MBA)^45^	Mine bomb explosion	2013
Social Based Algorithm (SBA)^46^	Human	2013
Grey wolf optimizer (GWO)^5^	Hunting nature of grey wolf	2014
Water wave optimization^47^	Water waves	2015
Moth-flame Optimization algorithm (MFO)^48^	Navigation method of moths in nature	2015
Optics Inspired Optimization (OIO)^49^	Law of reflection	2015
Whale Optimization Algorithm (WHO)^28^	Hunting behavior of Whale	2016
Dragon fly Algorithm (DA)^50^	Swarming behaviours of dragon flies in nature	2016
Kidney-inspired optimization algorithm^51^	Kidney process in the human body	2017
Grasshopper Optimisation Algorithm^52^	Grasshopper Swarms	2017
Class topper optimization (CTO)^4^	Student learning behaviour	2018
Kho-Kho optimization^53^	Indian Kho-Kho game	2020
Atomic orbital search^24^	Quantum mechanics principle	2021
Planet Optimization Algorithm^10^	Gravitational law of Newton	2022
Bottlenose Dolphin Optimizer^30^	Feeding nature of bottlenose dolphin	2022
Criminal search optimization^34^	Criminal identification technique based	2022

## Contribution and novelty of the study

The core of this paper is to establish a nature-inspired optimizer named the Human Conception Optimizer (HCO). Some unique features of the human conception process are utilized to develop the algorithm to solve any optimization problems. A method of generating healthy populations at the start of the HCO algorithm is modelled by replicating the concept of sperm selection by cervical gel according to the fitness of each one. A probability function is defined for this purpose. The probability function is formulated by considering a fraction of sperm (position of solutions or searching agents) lies between the best and worst positions in the population. During the generation of a healthy population, the concept of possible egg position in either ovary is also utilized. Thus, the initial generation of the population will also be based on the best combination of a randomly generated search variable and its oppositional directional (sperm positions) based search variable. Thus, the initial sperm positions (positions of solutions or searching agents) in the healthy population are already formed with the possible best direction where global solution may exist. Moreover, sperm oriented far away from the global solution or those that are towards the opposite direction of the global solution are ignored at the initial stage. Thus, the optimal solution can be searched within a healthy population with the possibility of getting the best solution quickly. Therefore, the issue of random initialization of the position of the search variables, which may be in different directions or far away from the global solution, is avoided in the HCO algorithm. Such a velocity profile will balance exploration and exploitation based on the fitness of the best sperm cell (position of search variable) and the fitness of the average sperm cells in an iteration. This will happen during the updating of positions of search variables (sperm cells). A hyperactivation function is also formulated by replicating the concept of flagellar oscillation during the hyperactivation stage of a sperm fertilizing an egg in the Fallopian tube. This function will help the algorithm escape from the local optimal solution.

## Human conception optimization algorithm

In this section, the inspiration and the mathematical modeling of the Human Conception Optimizer are explained in detail.

### Inspiration

**Figure 1:**
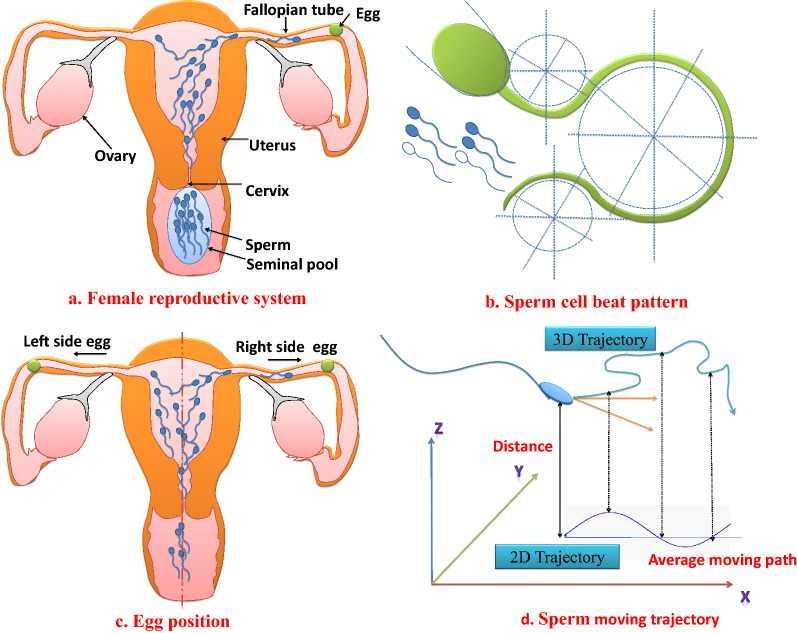
Human sperm movement. (**a**) Sperm cells movement in female reproductive system. (**b**) Sperm cells beat pattern. (**c**) Egg position in either side of fallopian tube. (**d**) Sperm cells moving trajectory.

Human conception happens when a healthy sperm cell meets the egg in the Fallopian tube^54^. The process begins with millions of sperm released into the female reproductive tract. All sperm cells compete to fertilize a single egg as presented in the Fig. 1a. In general, a single sperm is able to fertilize the egg in the Fallopian tube. Among the millions of sperm, a population of the most capable sperm can enter the door of the cervix. The cervical fluid called mucus, helps the spermatozoa swim through the uterus and the fallopian tube. Cervix filters out the liquid called semen which enclosed the sperm cells released into the vagina. Sperm uses a variety of mechanisms as they travel to the egg^55,56^. The method of sperm meeting egg for successful fertilization is explored in detail below.

#### Sperm fertilization bio-mechanism

Human conception occurs when a sperm cell is able to meet a mature egg, interact, and fuse in the female reproductive system^57^. Initially, sperm takes a random position in the vagina and stay inside the fluid called semen. According to the fitness of sperm, a swarm of the fittest sperm cells is able to enter the cervix. During their journey to the egg, sperm perform several outstanding navigational tasks. The sperm tail (flagellum) aids sperm swimming towards the egg by creating an irregular and oscillating beat pattern, as shown in Fig. 1b. While balancing the moment of force caused by flagellum motion, the cell head rotates and exerts force against the cervical fluid to move forward.

Sperm cells move with different hydrodynamic modes (such as typical, helical, hyperactivated or chiral ribbons) on the basis of environmental conditions such as temperature and viscosity inside the female reproductive system^58^. Sperm can collect physical and chemical information to identify the egg in the female genital system with the help of some mechanism, such as^59,60^:

- Rheotaxis-sperm orientation against the fluid to move upstream.
- Thermotaxis-sperm sense temperature variation in the reproductive system. It swims against a temperature gradient in a higher temperature zone near oviduct.
- Chemotaxis-the movement of cells up to a concentration gradient of chemoattractant. Sperm move toward increasing chemical concentration.

Chemotaxis was suggested in the literature as an active sperm guidance mechanism^61^. Sperm can sense the change in liquid concentration in the uterus. In thermotaxis, sperm move toward a higher temperature in the female reproductive system. The contractions of mucus in the female reproductive zone may also guide the sperm towards the egg.

Active sperm use a stroke called hyperactivation to cross the barrier of cumulus cells surrounding the egg. A fraction of sperm is able to become hyperactive. The flagellar beats of hyperactivated sperm have high curvature and a wider amplitude, leading to a highly active motility. Such a pattern of hyperactivity may create forces to facilitate sperm detachment and migration. The sperm have to pass another barrier called zonal pellucida (a layer of egg). The sperm cells undergo a process called the acrosome reaction, an enzyme deposited at the head of the sperm. It helps to break the zonal pellucida barrier to fertilise the egg^62^.

Among millions of sperm cells, only a single sperm cell is able to fertilize the matured egg in the challenging environment of the female reproductive system. The complete process is so challenging and unique that it motivates us to utilise the selection principles of winner sperm to develop a nature-inspired metaheuristic algorithm. In the next section, the detailed modelling of the proposed algorithm is discussed.

### Modeling of Human Conception Optimizer (HCO)

In this section, the biological principles of human conception are mathematically presented to develop the HCO algorithm. Generally, a set of natural facts and assumptions are considered to formulate the HCO algorithm. The concept of HCO is summarized as follows:

- After being released on the vagina, sperm cells enter the cervix, where their journey begins in a hostile environment. Only healthy sperm cells can enter the uterus and fallopian tubes^58^ (Fig. 1a). In a fertile female, either the right or left ovary produces a mature egg for fertilization as shown in Fig. 1b. The mucus fluid in the uterus helps sperm cells swim towards the egg^63^. This concept will be used to find a suitable initial fittest population from a randomly generated population of the initial positions of sperm cells or search agents. During the evaluation of sperm fitness, the possible position of the mature egg (global solution) will be examined by considering the right ovary as the place in a positive movement in the search area where the egg (global solution) may be found. The left ovary is considered as the place in a negative movement in the search area where the egg (global solution) may be found. The mucus fluid dynamics will be used to model the velocity of the sperm cells (sperm) to update their position during the exploration and exploitation stage of the proposed algorithm.
- The tail of the sperm creates a jerking like movement which helps the sperm move into the uterus. Sperm cells starts following the curvature path caused by flagellar movement to reach the egg^62^. This concept will be realised to model the sperm movement through a curvature tracking path during the searching procedure of the algorithm. At each iteration, the best position achieved by each sperm cell along the curved path will be evaluated and called the present best position or solution gained by each cell.
- The tail of sperm can sense the concentration of liquid in the reproductive system. According to that, it changes the position^62^. This sensing technique of liquid concentration in the reproductive system will be utilised to mimic the position update of sperm with respect to the best position of sperm achieved by any sperm cell in the population till the present iteration.
- Sperm cells overcome the barrier across the egg by a hyperactivation process. They have to pass another barrier called zonal pellucida. To pass such a barrier, sperm must undergo a process called acrosome reaction. This is an enzyme deposited at the top of sperm cells. It will break the zonal pellucida barrier, allowing sperm to penetrate the egg^60^. This concept will be used to overcome the local stuck problem of the algorithm.

The detailed modeling of the HCO algorithm is given below.

#### Initialization stage

During intercourse, millions of sperm cells are discharged into the female menstrual system. All cells try to enter the cervix. The liquid inside the cervix will allow only healthy cells to enter into the cervical tracking path. Therefore, there is a natural selection of initial healthy sperm cells where only fit cells can start the journey from the cervix towards the egg^64^. In HCO, each search agent resembles the position of the sperm cells. In any metaheuristic algorithm, the performance of a swarm-based optimization method depends on the initialization of the population. In HCO, the initial position of sperm cells will be generated randomly within a search space with a higher population size. From the initial population, a fitter population will be produced, which will follow the other steps of the proposed algorithm.

*Step 1: initial population generation* Let, there are ${``N''}$ number of sperm cells ejaculated into the vagina during intercourse. It indicates the same population number in the metaheuristic algorithm. The dimension of the population will depend on the optimization problem. The position of sperm cells is the position of sperm in the HCO algorithm. Each particle in the search space is the candidate of solutions for a particular optimization problem.

Let initial position of sperm cells (*X*) is defined as follows:

1 $$\begin{aligned} X=\left[ \begin{matrix} {{x}_{1}} \\ {{x}_{2}} \\ \vdots \\ \vdots \\ {{x}_{N}} \\ \end{matrix} \right] =\left[ \begin{matrix} x_{1}^{1} &{} x_{1}^{2} &{} \cdots &{} x_{1}^{d} \\ x_{2}^{1} &{} x_{2}^{2} &{} \cdots &{} x_{2}^{d} \\ \cdots &{} \cdots &{} \cdots &{} \cdots \\ x_{N}^{1} &{} x_{N}^{2} &{} \cdots &{} x_{N}^{d} \\ \end{matrix} \right] . \end{aligned}$$

In HCO, the initial positions of sperm cells are determined randomly as follows:

2 $$\begin{aligned} x_{i}^{j}=x_{{{i}_{\min }}}^{j}+{{r}_{1}}\times \left( x_{{{i}_{\max }}}^{j}-x_{{{i}_{\min }}}^{j} \right) , \end{aligned}$$

where ${i=1,2,\ldots ,N}$ and ${j=1,2,\ldots ,d}$; *N* is number of sperm cells or search agents inside the search space, *d* is the dimension of the problem, ${{r}_{1}}$ is the random number between 0 to 1, ${x_{i}^{j}}$ is the initial position of particle (sperm cells), ${x_{{{i}_{\max }}}^{j}}$ and ${x_{{{i}_{\max }}}^{j}}$ are the maximum and minimum limits of ${i\text {th}}$ sperm in ${j\text {th}}$ decision variable.

*Step 2: initial fitness evaluation-Modeling of egg position in the ovary* In a fertile female, either the right or left ovary produces a mature egg for fertilization, as shown in Fig. 1c. The right ovary is considered the place in positive direction in the searching area where the egg (global solution) exist. The left ovary is considered as the place in negative direction in the searching area where the egg (global solution) exist^64^. This concept is used in the proposed algorithm to check the solution to an optimization problem on both sides of the search space. During the evaluation of a solution candidate *x* for an assigned problem, the opposite solution of *x* may provide a better solution $x_op$. For example, if a solution of *x* is − 10 and the optimal solution is 40, then the opposite solution ($x_{op}$) is 10 and the distance of x from the optimal solution is 50. The distance between $x_{op}$ and the current best solution is 30. As a result, according to Ref.^64^, the opposite solution, $x_{op}$, is much closer to the global solution.

The algorithm first examines the fitness of all randomly generated initial search agents. The fitness values of all initial sperm (sperm cells) are defined as follows:

3 $$\begin{aligned} F(X)=\left[ \begin{matrix} f\left( {{x}_{{{f}_{1}}}} \right) \\ f\left( {{x}_{{{f}_{2}}}} \right) \\ \vdots \\ f\left( {{x}_{{{f}_{N}}}} \right) \\ \end{matrix} \right] =\left[ \begin{matrix} f\left( x_{{{f}_{1}}}^{1},x_{{{f}_{1}}}^{2},\ldots \right) \\ f\left( x_{{{f}_{2}}}^{1},x_{{{f}_{2}}}^{2},\ldots \right) \\ \vdots \\ f\left( x_{{{f}_{N}}}^{1},x_{{{f}_{N}}}^{2},\ldots \right) \\ \end{matrix} \right] , \end{aligned}$$

where *F*(*X*) is the fitness matrix with fitness value of all sperm (sperm).

*Position of opposite directional solution* The population of opposite directional solution will be calculated as follows:

4 $$\begin{aligned} {{X}_{oppo}}=a+b-{X}, \end{aligned}$$

where, *a* and *b* are lower and upper boundary of search agent respectively.

Fitness $F({{X}_{oppo}})$ of opposite directional population $({{X}_{oppo}})$ will be evaluated for an objective function based of the optimization problem.

Thus, the initial population based on egg position will be as follows:

5 $$\begin{aligned} \chi= \,& {} {X}\quad if\ F\left( {X} \right) >F\left( {{X}_{oppo}} \right) ; \end{aligned}$$

6 $$\begin{aligned}=\, & {} {{X}_{oppo}}\quad if\ F\left( {{X}_{oppo}} \right) >F\left( {{X}} \right) . \end{aligned}$$

##### Remark 1

In a fertile female, a mature egg is produced by either the right or left ovary for fertilisation every month during ovulation^61^. Typically, a single egg is released at a time. This concept can be modelled as for a single-objective optimization HCO algorithm. In some cases, more than one egg may be released, sometimes resulting in the conception of multiples (twins). This concept leads to the multiobjective HCO algorithm. To simplify, the present paper is discussed as a single objective HCO algorithm. The twins may be produced by fertilising a mature egg with two sperm cells. In the HCO algorithm, among two close solutions, the best one will be selected, ignoring the twin solution.

*Step 3: selection of healthy population* In the natural fertilization process, only healthy sperm cells can enter into the cervix to fertilize a mature egg. In HCO algorithm, the initial population size is taken as high as possible from where an initial fittest population will be selected according to a probability function. The fittest population will be allowed to follow the further steps of the proposed algorithm.

The best answer is assigned as the initial best solution (fittest sperm cell). The worst solution is also identified. The fitness of others will be compared with the fitness of the initial best with a probability of $(P_{fit})$. The probability of selecting the best population to move toward one of the best solutions is then calculated as follows:

7 $$\begin{aligned} {{{P}_{fit}}=[f(\chi _{{worst}})-f(\chi _{{best}})]\times {w}+f(\chi _{{best}})}, \end{aligned}$$

where *w* is a weight factor.

Therefore, the healthy population will be chosen as:

8 $$\begin{aligned} {{\chi }_{healthy}}={\chi }\ldots \ldots \ldots when F\left( {\chi } \right) \le {{P}_{fit}}. \end{aligned}$$

Thus,

9 $$\begin{aligned} {\chi _{healthy}}=\left[ \begin{matrix} {{\chi }_{{healthy}_1}} \\ {{\chi }_{{healthy}_2}} \\ \vdots \\ \vdots \\ {{\chi }_{{healthy}_n}} \\ \end{matrix} \right] =\left[ \begin{matrix} x_{{healthy}_{1}}^{1} &{} x_{{healthy}_1}^{2} &{} \cdots &{} x_{{healthy}_1}^{d} \\ x_{{healthy}_2}^{1} &{} x_{{healthy}_2}^{2} &{} \cdots &{} x_{{healthy}_2}^{d} \\ \cdots &{} \cdots &{} \cdots &{} \cdots \\ x_{{healthy}_n}^{1} &{} x_{{healthy}_n}^{2} &{} \cdots &{} x_{{healthy}_n}^{d} \\ \end{matrix} \right] , \end{aligned}$$

where ${{\chi _{healthy_i}}}$ is position of ${i\text{th}}$ healthy sperm, *n* is the size of fit population.

The fitness of initial fit population for an objective function depending on optimization problem will be as follows:

10 $$\begin{aligned} F({{\chi }_{healthy}})=\left[ \begin{matrix} f\left( {{{\chi }_{{healthy}_1}}} \right) \\ f\left( {{{\chi }_{{healthy}_2}}} \right) \\ \vdots \\ f\left( {{{\chi }_{{healthy}_n}}} \right) \\ \end{matrix} \right] =\left[ \begin{matrix} f\left( {x}_{{{healthy}_1}}^{1},{x}_{{{healthy}_2}}^{2},\ldots \right) \\ f\left( {x}_{{{healthy}_2}}^{1},{x}_{{{healthy}_2}}^{2},\ldots \right) \\ \vdots \\ f\left( {x}_{{{healthy}_n}}^{1},{x}_{{{healthy}_n}}^{2},\ldots \right) \\ \end{matrix} \right] , \end{aligned}$$

where ${F({{\chi }_{healthy}})}$ is the fitness matrix with fitness value of all healthy sperm cells (sperm).

The healthy or fit population will be used as the fittest initial population to search for the best solution for an optimization problem. In HCO, this step to find the fittest population from the initial randomly generated population will be done only once.

**Algorithm 1: Pseudo-code of HCO for generation of initial healthy population**

1. **Input:** Set population size of sperm position, other constants.
2.              $/*$ ***Generate initial random particle*** $*/$
3. Generate initial population for each variable randomly within a range of search space by using (2).
4.              $$/*$$ ***Evalute fitness*** $$*/$$
5. Evaluate fitness ${f(x_i)}$ of each particle (${x_i}$) for each variable with an objective function for a optimization problem. Calculate fitness ${f(x_{i_{oppo}})}$ with opposite directional sperm ${x_{i_{oppo}}}$.
6.     if $${{f(x_i)}>{f(x_{i_{oppo}})}}$$
7.     Select $${x_i}$$
8.     else
9.      Select $${x_{{i}_{oppo}}}$$
10.     *end* *if*
11.              $/*$ ***Select initial best and worst particle*** $*/$
12. Find the best fitness ${f_{best}(x)}$ and worst fitness (${f_{worst}(x)}$) from the fitness matrix (10).
13. Derive the probability function using (7).
14.      if $${f({{\chi }_{i}})\le {{P}_{fit}}}$$
15.      Update fit population using (8).
16.      else discard and check for next healthy sperm.
17.      *end* *if*
18. **Output:** Initial healthy population

##### Remark 2

Sperm orientation can be a replica of particle orientation. Some sperm may be towards the global solution and some may be alongside the boundary of the search space. Some of them may be in the opposite direction of the global solution. In HCO, the initial fittest population is chosen with sperm (position of search agents) oriented towards the egg (best initial solution).

#### Sperm movement modeling-Position update of particle

The male reproductive cell, sperm, has a single flagellum or a tail. To achieve fertilization, sperm needs to move up the oviduct. The sperm’s tail produces a distinctive, jerky motion that pushes the head of the sperm backward and sideways while simultaneously propelling the sperm forward. The cells migrate through the fluid in the cervix by moving backwards and sideways. The sperm cell is aided in its journey toward the egg by this combination of actions. They can’t swim backwards due to the nature of flagellar movement. The moving trajectory of the sperm cell is shown in the Fig. 1d.

Human sperm use various sensing mechanisms to gather physical or chemical signals to spot the egg. During the fertilisation process, sperm cells move along the narrow cervical tracking path towards the oviduct. Mucus in the cervix helps sperm move through the uterus and oviducts^62^. There are three types of sperm swimming guidance mechanisms: thermotaxis (based on temperature gradient), rheotaxis (swimming against a fluid flow), and chemotaxis (based on chemoattractant concentration gradient)^55^. Sperm cells will move against the mucus flow, which is a rheotaxis mechanism. They assume the egg position based on the concentration of liquid change near the egg. In HCO, the rheotaxis mechanism of sperm guidance towards the egg is used to find the velocity of sperm in fluid against the flow. The flagellar asymmetric movement is taken as a sinusoidal curvature path in the HCO algorithm.

##### Velocity profile

The human spermatozoa can sense a flow of liquid and change the direction of their path against the flow. It performs positive rheotaxis and orients itself against an oncoming flow. Mucus flow (like as a sperm cell flow in fluid) can be described by the Poiseuille profile, where the speed increases quadratically with the distance to the compartment boundary. The Poiseuille profile is used to find the speed of the sperm cells. It tells how fast the sperm cells are moving at each point within the uterus^65,66^.

**Figure 2:**
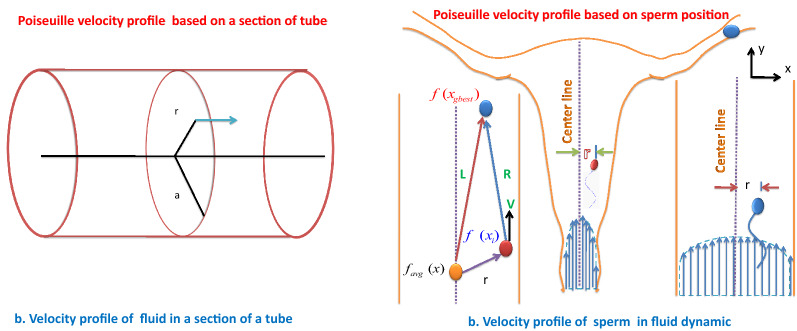
Sperm velocity profile: (**a**) a section of tube of radius (a), Velocity of fluid at a distance r from the center of the tube. (**b**) Sperm velocity profile based on.

In HCO, the Poiseuille velocity profile is used to model the velocity of sperm to update their position. The Poiseuille velocity profile for sperm movement in the female reproduction tracking path is shown in Fig. 2b. To model the Poiseuille velocity tracking profile, the fitness matrix (10) will be used.

##### Poiseuille velocity profile

The velocity profile shows the amplitude of velocity according to the position of a particle in a fluid. According to the Poiseuille velocity profile, the velocity at a point, called specific radius (*r*) in the fluid can be calculated by measuring the distance of the point from the centre of the tube, as graphically shown in Fig. 2a. At the specific radius (*r*), the velocity is formulated as^65,66^:

11 $$\begin{aligned} V =\frac{P \left( {{a}^{2}}-{{r}^{2}} \right) }{4\eta L}, \end{aligned}$$

where *P* is the pressure difference, *L* is length of a pipe with radius *a*, ${\eta }$ is dynamic viscosity.

In HCO, the fitness sperm is used to mimic the velocity profile. The velocity of a sperm in the current iteration is calculated by taking the sperm’s current position ${{{\chi }}_{i}}$ in the healthy population and multiplying it by its fitness ${f\left( {{{\chi }}_{i}} \right) }$. The centre of the flow resembles the average position of sperm with a fitness of (${f({\chi }_{avg})}$). The fitness level of the present global best position is ${f({\chi }_{best})}$.

Steps to mimic Poiseuille velocity tracking profile for sperm’s velocity modeling:

- Assign the initial best fitness value of a sperm cell for a given optimization problem with a fitness function in a iteration as ${f({\chi }_{best})}$.
- Calculate the average fitness ${f({\chi }_{avg})}$.
- Calculate the velocity of ${i\text{th}}$ sperm cell with the fitness value ${f(x_i)}$as follows:
  12 $$\begin{aligned} {{\nu }_i} =\frac{\gamma \left( {{R}^{2}}-{{r}^{2}} \right) }{4\eta L}, \end{aligned}$$

where ${R=f\left( {{{\chi }}_{best}} \right) -f\left( {{{\chi }}_{i}} \right) }$, ${r=f\left( {{{\chi }}_{avg}} \right) -f\left( {{{\chi }}_{i}} \right) }$, ${L=f\left( {{{\chi }}_{best}} \right) -f\left( {{{\chi }}_{avg}} \right) }$, ${{\nu }_i}$ is velocity of ${i\text{th}}$ sperm cell, ${f\left( {{{\chi }}_{avg}} \right) }$ is the average health of the population, ${f\left( {{{\chi }}_{best}}\right) }$ is the health of best solution (optimal position), ${\eta }$ is a constant generated with random value in the range of 0 to 1, and ${\gamma }$ is a random number between 0 and 1.

The vector diagram of velocity profile of sperm cells is also shown graphically in Fig. 2b.

##### Velocity update

After entering into the cervix, the sperm cells grabbed an initial velocity in the cervical fluid. In HCO, sperm initial velocity is modelled according to the Poiseuille velocity tracking profile as presented in Fig. 2b. The position of a sperm cell in the current iteration will be compared with its previous position, and the best one will be assigned as the present best solution $(S_{p_{best}})$ for the sperm cell. In the healthy population, one sperm cells achieved the best position among all in an iteration and will be treated as the global best solution $(S_{g_{best}})$ in that iteration. The sperm cellc will move along a sinusoidal path, resembling the nature of the sperm movement in a curvature path with the updated velocity.

In the search space the velocity of sperm will be updated as follows:

13 $$\begin{aligned} {{\vec {V}}_{i}}\left( j+1\right) ={w_1}\times ({{\vec {V}}_{i}}(j)+{{\nu }_i}(j))+{C_1} \times {A_1}\times {\sin \left(2\pi \frac{j}{j_{max}}\right)}+{C_2}\times {A_2}\times {\sin \left(2\pi \frac{j}{j_{max}}\right)}, \end{aligned}$$

where ${{A_1}}$ is ${(S_{p_{best}}-S_i)}$; ${{A_2}}$ is ${(S_{g_{best}}-S_i)}$; ${C_1}$ is a constant; ${C_2}$ is a constant.

##### Position update

Along the curvature path, the position of sperm will be updated in HCO as follows:

14 $$\begin{aligned} {{\vec {{\chi }}}_{i}}\left( j+1 \right) ={{\vec {{\chi }}}_{i}}\left( j \right) + {{\vec {V}}_{i}}(j+1), \end{aligned}$$

where ${{{\vec {{\chi }}}_{i}}(j)}$ is the position of ${i\text{th}}$ sperm at ${j\text{th}}$ iteration, ${{{\vec {V}}_{i}}(j)}$ is the velocity of ${i\text{th}}$ sperm at ${j\text{th}}$ iteration.

**Algorithm 2: Pseudo-code of HCO for update sperm position**

1. **Input:** Healthy population of initial sperm positions, define other constants
2.              $/*$ ***Generate initial healthy population of sperm positions*** $*/$
3. Generate initial healthy population of sperm position for each variable according to Algorithm 1.
4.              $/*$ ***Evalute fitness function*** $*/$
5. Evaluate fitness ${f({\chi }_i)}$ of each sperm (${{\chi }_i}$) for each variable with an objective function for a optimization problem.
6. Identify average fitness of sperm (${f({\chi }_{avg})}$) in the population, fitness of best sperm (${f({\chi }_{best})}$).
7. Identify the best sperm (global solution) $(S_{g_{best}})$ achieved at present iteration. Also, identify the current best position of each sperm $(S_{p_{best}})$ at current iteration.
8.              $/*$ ***Evalute velocity of sperm*** $*/$
9. Evaluate velocity of each sperm in the healthy population using (12).
10.              $/*$ ***Update velocity of sperm*** $*/$
11. Update velocity of sperm using (13).
12.              $/*$ ***Update position of sperm*** $*/$
13. Update the position of each sperm using (14).
14.     Repeat step 5 to 13 till the termination criterion reached or maximum number of iteration.
15. **Output:** Fittest sperm or global solution.

**Figure 3:**
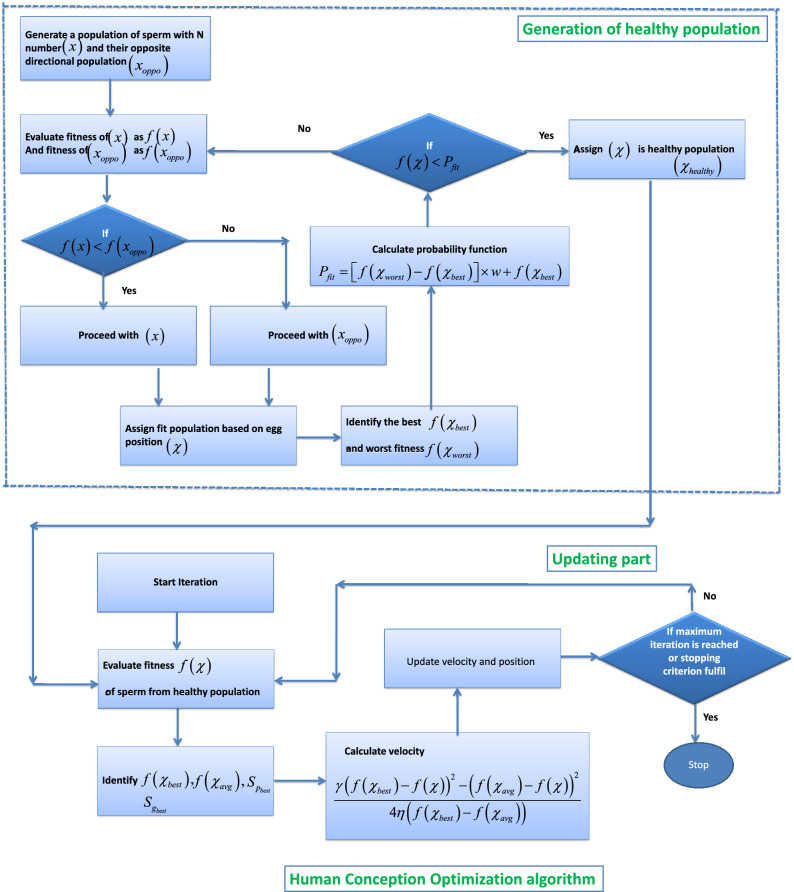
Flowchart of proposed HCO algorithm.

The flowchart of the proposed algorithm is presented in Fig. 3.

#### Sperm hyperactivation-local optimal solution avoidance

##### Sperm hyper-activation

In human conception, sperm cells conform a obstacle of cumulus cells around the egg. Before reaching the egg, the sperm cells are often trapped in epithelial cells in the fallopian tube. They are rendered inert unless they undergo hyperactivation^67^. To cross this barrier of cumulus, the sperm cells must use a very specific stroke called hyperactivation. It is characterised by an asymmetrical flagellar beat pattern which rises to a whip-like movement of the flagellum that can produce circular figure-eight swimming trajectories. The change in motion and force of the tail movement in the trajectory enable the sperm to escape from the epithelium^67^.

##### Modeling of hyper-activation

In HCO, the concept of sperm hyperactivation process will be adapted when the best solution is found stuck in a position for a long time before reaching termination criteria. The position of the hyperactivated particle will be compared with the best solution achieved before the hyperactivation process. Among the hyperactivated solutions and the non-hyperactivated solutions, the best one will be assigned as the current global solution for the population. To model the hyperactivation process, eight (8) shaped beat patterns are used. The new position of the best hyperactivation particle is modeled as follows:

15 $$\begin{aligned} x_{hyperactivated}(j)={{x}^{gbest}}(j)\times (1+\times \left\{ \sin \left( 2\times \pi \times {m_1} \right) \times \cos \left( 2\times \pi \times {m_2} \right) \right\} ; \end{aligned}$$

16 $$\begin{aligned} {{x}_{globalbest}}\left( j \right) =\left\{ \begin{matrix} {{x}_{hyperactivated}}\left( j \right) ,\quad \quad if\ \ f\left( {{x}_{hyperactivated}} \right)>f\left( {{x}^{gbest}} \right) \\ {{x}^{gbest}}\left( j \right) ,\quad \quad \quad \quad \quad if\ \ f\left( {{x}^{gbest}} \right) >f\left( {{x}_{hyperactivated}} \right) \\ \end{matrix} \right. \end{aligned}$$

where ${{x}_{globalbest}(j)}$ is the global best solution at ${j\text{th}}$ iteration, ${x_{hyperactivated}(j)}$ is hyperactivated best solution at ${j\text {th}}$ iteration. It will be used only when the global best solution get stuck at same position for more than two iteration.

### Features of HCO algorithm

HCO provides some advantages which makes it unique from some others algorithm. Some spatial features are as follows:

- *Concept of healthy initial population* The HCO algorithm replicates the concept of sperm selection by the female reproductive system to allow them in the cervix and the position of the egg in either of the ovaries. The initial population in the HCO algorithm is not assigned directly to a randomly generated initial sperm within a search space. In this algorithm, a healthy population is generated at the initial stage by neglecting the sperm in the population oriented so far from the optimal position. Using the concept of egg position in the right or left ovary in the Fallopian tube, the fittest of all randomly generated sperm is evaluated along with the fitness of their opposite directional sperm in the search space. Thus, the healthy population will be based on the best possible solution in the positive or negative direction in the search space. The healthy population will ensure the best initial fit population within which the optimal solution will be found by the algorithm. Healthy populations will include initial positions of sperm based on their initial fitness and the best side of the position of the mature egg (global solution) by checking a sperm position and its opposite distortional position.
- *Velocity update based on Poiseuille Velocity profile* During the updation of the velocity of sperm cells, the position-based velocity profile is used, called the Poiseuille Velocity profile. The advantage of using such a velocity profile in the HCO algorithm is that the velocity of each sperm or search variable at an iteration will be calculated with the fitness value of the best position of a sperm or search variable in that iteration along with the average fitness in the population. Therefore, a good balance can be maintained between the exploration and exploitation stages of the algorithm.
- *Hyperactivation for local optima avoidance* Like sperm’s hyperactivation process to fertilize egg, a hyperactivation function is used in the HCO algorithm to avoid local solution trapping problems.

## Numerical test of HCO algorithm-benchmark functions

A metaheuristic algorithm must have some capabilities to solve complex optimization problems. An optimizer must exhibit a good balance in exploration and exploitation stages, local optima avoidance, and smooth convergence capability. To check the achievement of the HCO algorithm, two suites of test functions are taken in the study, such as 23 numbers of classical test functions from the CEC 2005 special session and ten number of 30 and 500 dimensional benchmark or test functions from the CEC-2020^24^.

### Case study 1: CEC 2005 benchmark function (BMF)

In this section, the response of the HCO algorithm is verified with CEC 2005 BMFs^24^. Such functions are minimization functions. They can be grouped as: unimodal, multimodal, and fixed-dimensional multimodal. The details of such BMFs can be found in the CEC 2005 technical report^24,27^. The termination of the algorithm is set at a fixed iteration. The other parameters for the HCO algorithm are presented in Table 2. The LabVIEW©2015 platform is used for the simulation purposes of the algorithm. This algorithm is executed several times for each reference function. After several tests, the average and standard deviation (*SD*) of each BMF are examined. The convergence performance for each BMF with the HCO method is carried out and compared with PSO^6^, CTO^4^, GWO^5^, WHO^28^, and SFO (Sailfish Optimizer)^29^.

**Table 2:** Parameters of HCO algorithm.

Parameter	Value
Population size	500
Iteration number	500
${P_{fit}}$	0.65
${\lambda }$	Random number (0 to 1)
w	0.1
${A_1}$ and ${A_2}$	Random number (2 to 4)

**Figure 4:**
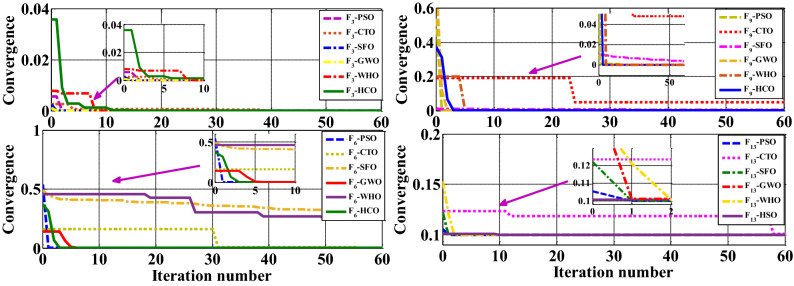
Comparison of convergence performance of HCO for CEC2005.

For each BMF, the population size is considered as 500, and the maximum iteration is 500. Other constants of the HCO algorithm are tabulated in Table 2. The HCO algorithm is executed 30 times, with 500 iterations for each function. For analysis purposes, the average and *SD* of objective values are examined. The output of the BMFs is presented in the Table 3. There is a single global optimum point for unimodal functions (${F_1}$ TO ${F_7}$)^27^. By looking at the Table 3, it can be seen that HCO performed better for ${F_1}$, ${F_2}$, ${F_6}$ and ${F_7}$ than CTO, PSO, and SFO. For function ${F_4}$, HCO is better than CTO, PSO. For function ${F_5}$, HCO is found better than SFO, PSO. For function ${F_6}$, HCO is found better than CTO, SFO. For the function ${F_3}$, GWO is found better than HCO. For the function ${F_6}$, HCO is found better than GWO as found in Fig. 4. For the functions ${F_3}$ and ${F_6}$, HCO is found better than WHO as found in Fig. 4.

**Table 3:** Convergence performance comparison for CEC 2005 benchmark function.

Function	SFO^29^		PSO^6^		CTO^4^		GWO^5^		WHO^28^		HCO	
	Ave	Std	Ave	Std	Ave	Std	Ave	Std	Ave	Std	Ave	Std
${F_1}$	6.59E−28	6.34E−05	0.000136	0.000202	3.50E−19	6.59E−19	6.59E−28	6.34E−05	8.2E−14	5.9E−14	0	0
${F_2}$	7.18E−17	0.029014	0.042144	0.045421	5.44E−10	5.96E−10	7.18E−17	0.02901	1.5E−09	9.9E−10	4.23E−48	3.65E−63
${F_3}$	4.95196E−06	0.000110343	2.26299E−05	0.000356831	7.1739E−05	0.000295386	2.43054E−06	5.22709E−05	6.8E−11	7.4E−11	0.000194706	0.002313515
${F_4}$	5.61E−07	1.315088	1.086481	0.317039	4.52507	1.09056	5.61E−07	1.31508	0	0	0.000321656	0.002229746
${F_5}$	26.81258	69.90499	96.71832	60.11559	1.30E−19	2.11E−19	26.8126	69.9049	0	0	0.772516484	0.017427571
${F_6}$	0.09742269	0.128767789	0.001110401	0.024122691	0.005434	0.038755873	0.001254672	0.012670857	0	0	0.001050433	0.021926755
${F_7}$	0.002213	0.100286	0.122854	0.044957	0.005434	0.002232	0.00221	0.10028	0.00463	0.0012	0.000464401	0.002356391
${F_8}$	− 6123.1	− 4087.44	− 4841.29	1152.841	− 2791.7	330.711	− 6123.1	-4087.44	− 11080.1	574.7	− 418.983	31.20867554
${F_9}$	0.00110267	0.002217182	8.73976E−07	9.6584E−06	0.013548005	0.042302315	0.00186388	0.036574417	69.2	38.8	0.000146449	0.002534135
${F_{10}}$	1.06E−13	0.077835	0.276015	0.50901	1.5035	0.651061	1.06E−13	0.077835	9.7E−08	4.2E−08	1.69024706	0.036404351
${F_{11}}$	0.004485	0.006659	0.009215	0.007724	0.211515	0.018247	0.00448	0.006659	0	0	0.001641388	0.014162738
${F_{12}}$	0.053438	0.020734	0.006917	0.026301	6.08416	4.80515	0.05344	0.020734	7.9E−15	8E−15	− 0.916816662	1.05578E−05
${F_{13}}$	0.100043904	0.000981365	0.100011116	0.000238848	0.10226571	0.006217516	0.65446	0.004474	5.1E−14	4.8E−14	0.10001064	8.77149E-05
${F_{14}}$	0.665779	8.22388E−15	2.663115884	31.54900921	0.665779	8.22388E−15	4.0425	4.25289	0.998004	3.3E−16	0.665779	8.22388E−15
${F_{15}}$	0.000337	0.000625	0.000577	0.000222	0.000434	0.000238	0.00034	0.000625	4.5E−14	0.00033	0.051513612	8.4432E−06
${F_{16}}$	− 1.03163	− 1.03163	− 1.03163	6.25E−16	− 1.03136	0	− 1.03163	− 1.03163	− 1.03163	3.1E−13	− 1.030402677	0.014350901
${F_{17}}$	0.545282286	0.127323904	0.398208716	0.006995143	0.462937336	0.011283894	0.3978	0.3978	3.397887	9.9E−09	0.398564315	0.005786036
${F_{18}}$	3.000028	3	3	1.33E−15	3	0	3.000028	3	3	2E−15	3.0199801	0.184129826
${F_{19}}$	− 3.86263	− 3.38278	− 3.86278	2.58E−15	− 3.8794	2.56E−16	− 3.8626	− 3.38278	− 3.86288	− 3.38217	− 3.86178	2.58E−15
${F_{20}}$	− 3.28654	− 3.25056	− 3.26634	0.060516	− 3.3222	0	− 3.2865	− 3.2505	− 3.18654	− 3.05056	− 7.74807	6.84582E−14
${F_{21}}$	− 10.1514	− 9.14015	− 6.8651	3.019644	− 3.86903	1.36967	− 10.1514	− 9.1402	− 10.1532	0.0000025	− 5.3977697	6.7082E−06
${F_{22}}$	− 10.4015	-8.58441	-8.45653	3.087094	− 10.4029	1.26E-15	− 10.4015	−8.584	-10.4029	3.9E−07	− 10.4029	3.9E−07
${F_{23}}$	− 10.5343	-8.55899	− 9.95291	1.782786	− 10.5364	1.26E−15	− 10.5343	− 8.5589	− 10.5364	1.9E−07	− 6.18343928	8.22295E−06

The multi-modal functions such as ${F_8}$ to ${F_{13}}$^27^ exhibit multiple local optimal. The exploration feature of an optimization method may be verified with multimodal functions. By analyzing the Table 3, HCO performed better than PSO, CTO, and SFO for ${F_{11}}$ to ${F_{13}}$. Other multimodal functions also offered better performance compared to PSO, CTO, and SFO. For function ${F_8}$, the minimum value of ${F_8}$ as specified by CEC2005^24^ is found by the HCO algorithm where others are not able to find the same. For function ${F_9}$, HCO is better than CTO, SFO. For some fixed dimensional functions such as ${F_{19}}$ to ${F_{22}}$^27^, HCO is superior to SFO, CTO, and PSO. The convergence curve of the HCO algorithm for some BMFs is analyzed. Some BMFs such as ${F_{3}}$, ${F_{6}}$, ${F_{9}}$, ${F_{13}}$, ${F_{14}}$ and ${F_{17}}$ are considered to show the convergence characteristic of the HCO algorithm in Fig. 4. For the function ${F_9}$ and ${F_{13}}$, GWO is found better than HCO as found in Fig. 4. For the functions ${F_9}$ and ${F_{13}}$, HCO is found better than WHO as found in Fig. 4.

### Case study II: CEC 2020 benchmark function

**Figure 5:**
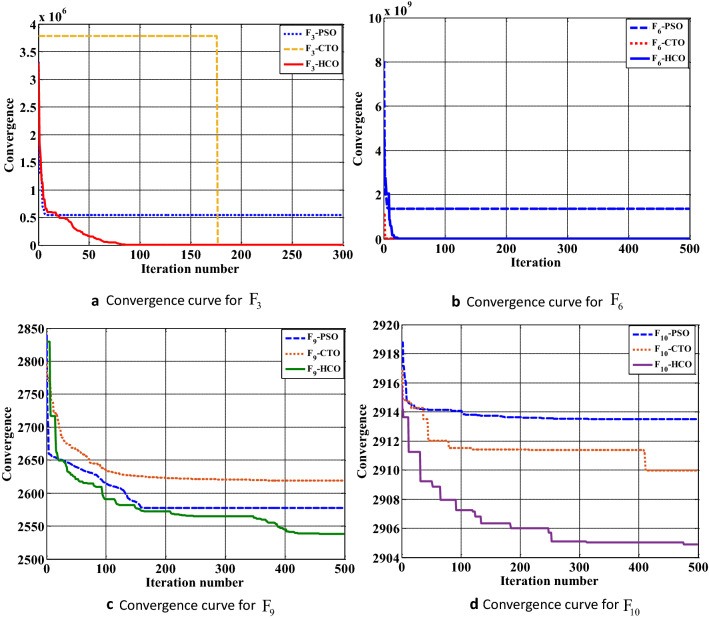
Convergence performance of HCO for 30D CEC2020 benchmark function: (**a**) Convergence graph for ${F_3}$ benchmark function. (**b**) Convergence graph for ${F_6}$ benchmark function. (**c**) Convergence graph for ${F_9}$ benchmark function. (**d**) Convergence graph for ${F_{10}}$ benchmark function.

**Figure 6:**
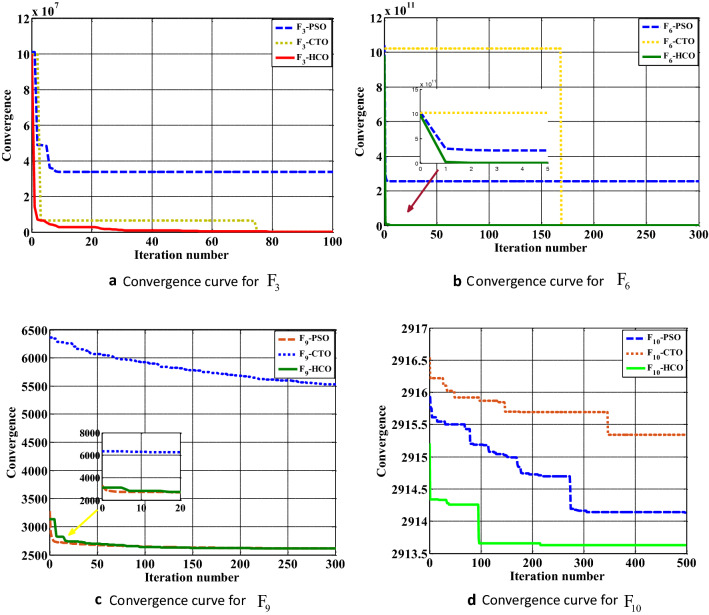
Convergence performance of HCO for 500D CEC2020 benchmark function: (**a**) Convergence graph for ${F_3}$ benchmark function. (**b**) Convergence graph for ${F_6}$ benchmark function. (**c**) Convergence graph for ${F_9}$ benchmark function. (**d**) Convergence graph for ${F_{10}}$ benchmark function.

The HCO algorithm is verified with CEC-2020 BMFs to explore the achievement of the algorithm in terms of exploration, exploitation, convergence, and local optima avoidance. It includes unimodal, multi-modal, hybrid, and composite functions to validate the proposed algorithm. Each function has been tested with two conditions: with a 30 variable based optimization problem and another one with a 500 variable problem, and simulated 20 times in LabVIEW©2015 platform. The results in terms of average value and standard deviation are computed after 20 tests run in each of the MBF. The achievements of the HCO algorithm for each MBF are compared with some existing methods as reported in the literature, such as PSO^6^, CTO^4^. For simulation purposes, each function is tested in a population of 50 sperm and run for 500 iterations for 30 dimensional problems and 500 dimensional problems. The convergence performance of the HCO for some selected 30 dimensional CEC-2020 benchmark functions is shown in the Fig. 5. In most of the cases of 30 dimensional CEC-2020 benchmark functions, HCO performed better than the available algorithms as tabulated in Table 4. The convergence performance of the HCO for some selected 500 dimensional CEC-2020 benchmark functions is shown in the Fig. 6. In most of the cases of 500 dimensional CEC-2020 benchmark functions, HCO performed better than any available algorithms as tabulated in Table 5.

**Table 4:** Convergence performance of HCO for 30 dimensional CEC 2020 benchmark functions.

Function	PSO^6^		CTO^4^		HCO	
	Ave	Std	Ave	Std	Ave	Std
${F_1}$	26881951200	5648656623	18410919163	39825804953	2328904813	9287733381
${F_2}$	7418.48304	211.6382903	3376.88648	2942.944784	5812.17144	916.1663569
${F_3}$	553631.542	136920.4716	1338036.873	1808228.112	63862.4367	235631.2497
${F_4}$	7.79934E+14	1.20314E+16	7.51269E+14	9.67911E+15	1.42579E+15	2.42858E+16
${F_5}$	35321691.2	16488340.61	2798099.436	22847917.82	1778318.217	12016004.07
${F_6}$	1372479800	344836668.7	7981480.045	68808804.21	55315895.78	367878725.3
${F_7}$	149909717.5	353492605.4	1097011670	1927553881	8759421.951	60018549.34
${F_8}$	2519.72106	358.0909202	2428.44478	321.9716397	2485.38706	622.6405186
${F_9}$	2593.23288	2622.252949	2632.99216	28.46593127	2577.94732	44.19822566
${F_{10}}$	2913.74316	0.562921465	2906.35766	1.978006587	2911.45742	1.11123915

**Table 5:** Convergence performance of HCO for 500 dimensional CEC 2020 benchmark function.

Function	PSO^6^		CTO^4^		HCO	
	Ave	Std	Ave	Std	Ave	Std
${F_1}$	7.53155E+11	1.51623E+11	1.0728E+12	1.40557E+12	13025933321	1.34781E+11
${F_2}$	169281.614	0	8786.7941	16928.40751	17593.2522	7216.447467
${F_3}$	34301329.8	4457602.287	1548553.152	7973805.107	481592.3622	4593483.07
${F_4}$	8.16935E+17	3.28054E+18	2.97555E+17	3.83138E+18	1.07025E+17	1.71899E+18
${F_5}$	53823538200	326384053.5	16801500609	27308894986	247311424	2696128450
${F_6}$	2.57104E+11	35142505673	3.44456E+11	4.82546E+11	2088397677	43904983536
${F_7}$	217638E+6	32108985595.939	0.089441	0.04339	2407662922	50738603427
${F_8}$	7601.60744	17765.58749	12011.64214	17834.34609	4355.0323	6620.251597
${F_9}$	2628.8713	44.97289066	5668.80494	266.6878197	249.5	69.73859373
${F_{10}}$	2914.6585	0.528857761	3.89347E+11	5.4788E+11	2913.768224	0.270776361

*The use of non-parametric tests* is also tested HCO algorithm for the CEC2020 benchmark functions. Such tests have a wide variety of applications in literature^27^. In this paper, the Friedman and Wilcoxon signed test are carried out CEC 2020 benchmark functions as presented in Tables 6 and 7 respectively. The details of such methods are provided in Ref.^27^. In the Wilcoxon signed test, ${r^{+}}$ is the summation of ranks when the first method is better compared to second method, and ${r^{-}}$ is the opposite condition at a significant measure of ${\alpha = 0.05}$.

**Table 6:** Friedman test statistical data for 30D CEC2020 BMFs.

Function	PSO^6^		CTO^4^		HCO	
	Ave	Rank	Ave	Rank	Ave	Rank
${F_1}$	26881951200	1	18410919163	2	2328904813	3
${F_2}$	7418.48304	2	3376.88648	1	5812.17144	3
${F_3}$	553631.542	1	1338036.873	2	63862.4367	3
${F_4}$	7.80E+14	1	7.51E+14	2	1.43E+15	3
${F_5}$	35321691.2	1	2798099.436	2	1778318.217	3
${F_6}$	1372479800	1	7981480.045	2	55315895.78	3
${F_7}$	149909717.5	1	1097011670	2	8759421.951	3
${F_8}$	2519.72106	2	2428.44478	1	2485.38706	3
${F_9}$	2593.23288	1	2632.99216	3	2577.94732	2
${F_{10}}$	2913.74316	2	2906.35766	1	2911.45742	3
Total rank		13		18		29
Average rank		1.3		1.8		2.9
p-value			0.0012

**Table 7:** Wilcoxon-signed test for CEC2020 BMFs at significant stage ${\alpha =0.05}$.

Function	30D			500D		
	${r^+}$	${r^-}$	p-value	${r^+}$	${r^-}$	p-value
HCO versus PSO^6^	10	45	0.03754	0	55	.00256
HCO versus CTO^4^	25	30	0.40129	8	47	.0233

## HCO for engineering problems

In this section, the performance of the proposed HCO algorithm is validated for two constrained engineering optimization problems. In the first case, a over-current relay coordination based optimization problem is chosen. In this problem, the HCO is used to get an optimal setting of the over-current relays used in the protection scheme of a power distribution network. In the second case, an optimal PID controller is designed for the human respiratory ventilation system. In this problem, the proposed HCO algorithm is used to tune the PID controller for a blower type ventilator model.

### Case study 1: HCO for optimal over-current relay coordination problem in power distribution systems

**Figure 7:**
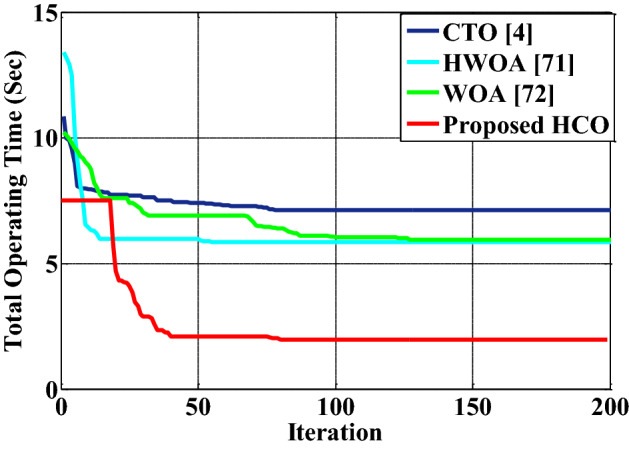
Convergence response curve of HCO for relay coordination problem.

In this optimization problem, optimal coordination is established among over-current relays used in a power distribution network to supply uninterrupted power. To validate the proposed HCO algorithm for such a real-world engineering problem, an IEEE 8-bus distribution network with 14 over-current relays is considered. It consists of 20 numbers of selectivity limitations at three phase fault. The range of relay time dial setting (TDS) is between 0.1 and 1.1 s. The co-ordination time interval is 0.3 second. Plug setting is between 0.5 and 2.5. The other specifications such as current transformer ratio, short circuit current at fault locations are taken from Ref.^68^. To get the optimal operating time of the protective relay system, the *PS* (plug setting) and *TDS* (Time Dial Setting) are optimized. The test system is executed with HCO in LabVIEW©2015 platform. The convergence performance for the relay coordination problem is shown in Fig. 7. In the Fig. 7, it can be observed that the algorithm started with a better initial value and converged fast towards a better solution compared to others. The optimal settings of relays found by the HCO algorithm for the IEEE-8 bus distribution system are presented in Table 8. A comparative analysis of the proposed method for the same system as discussed in^68^ with some existing results is carried out. For this purpose, some well-known methods, such as BBO-LP^69^, BIP^70^, HWOA^71^, WOA^72^, MWCA^73^ and SA-LP^74^ are considered for the same system and presented in Table 9. From the Table 9, the total relay operating time gained by the proposed algorithm is better than the existing results.

**Table 8:** Optimal relay setting for IEEE 8 bus distribution system with 14 number of over-current relay.

Prelay	TDS	PS	Srelay	TDS	PS
${R_1}$	0.0278465	1.90667	${R_1}$	0.232135	1.13722
${R_2}$	0.0497897	1.44066	${R_2}$	0.39036	1.44429
${R_3}$	0.31589	0.550752	${R_3}$	0.234753	1.98357
${R_4}$	0.123364	0.555611	${R_4}$	0.193875	1.58553
${R_5}$	0.05704	0.5308	${R_5}$	0.127873	0.898946
${R_6}$	0.0625788	1.1815	${R_6}$	0.181345	0.597147
${R_7}$	0.0373099	1.60793	${R_7}$	0.43253	0.536434
${R_8}$	0.0384561	1.07845	${R_8}$	0.216296	1.99503
${R_9}$	0.0737711	1.07065	${R_9}$	0.355793	0.52301
${R_{10}}$	0.0874095	0.569945	${R_{10}}$	0.340732	1.90873
${R_{11}}$	0.044051	0.540862	${R_{11}}$	0.395206	1.18428
${R_{12}}$	0.0519719	0.582733	${R_{12}}$	0.311382	1.58579
${R_{13}}$	0.0420051	0.548888	${R_{13}}$	0.351574	1.90349
${R_{14}}$	0.034316	0.9902	${R_{14}}$	0.318955	1.36453

**Table 9:** Comparison of operating time for IEEE 8 bus distribution system.

Method	BBO-LP^69^	BIP^70^	HWOA^71^	WOA^72^	MWCA^73^	SA-LP^74^	Proposed HCO
Operating Time (second)	8.7556	8.6944	5.8568	5.9535	6.4	8.4271	1.96

### Case study 2: HCO for PID controller design for human respiratory mechanical ventilator (MV)

In this engineering problem, the HCO algorithm is used as a tuning method for the proportional derivative integral controller (PID) used in the mechanical ventilator (MV) system used in the intensive care unit (ICU). The parameters of the conventional PID controller must be efficiently set such that the ventilator system can provide sufficient air to maintain a stable air-pressure in the lung system. The details of the mathematical modeling, and associated constraints MV are studied from Ref.^75^. For simulation purpose, the transfer function (${{G}_{1}}(s)$) of the patient-hose system with lungs compliance of 20 ml/mbar, and lungs airway resistance of 5 mbar s/l is expressed as follows^75^:

17 $$\begin{aligned} {{G}_{1}}\left( s \right) =\frac{0.5063{{s}}+5.063}{s+5.443}. \end{aligned}$$

The transfer function of blower system (${{G}_{2}}$ ) is taken as^75^:

18 $$\begin{aligned} {{G}_{1}}\left( s \right) =\frac{35530}{{s^2}+377s+35530}. \end{aligned}$$

As a desired breathing pattern, an unit pulse of period 2 is selected. The initial ranges of parameters of the PID controller (${{K}_{p}}$, ${{K}_{i}}$ and ${{K}_{d}}$ ) are considered from^27^ as follows:

1. $${1\le {K}_{p}\le 2}$$,
2. $${100\le {{K}_{i}}\le 200}$$,
3. $${0\le {{K}_{d}}\le 0.1}$$.

**Figure 8:**
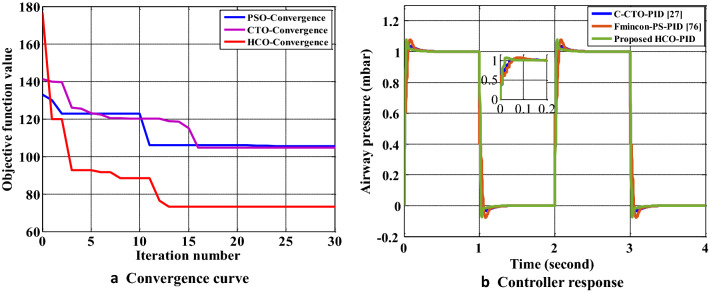
Performance of HCO for PID controller design for ventilator system: (**a**) Convergence graph. (**b**) Response of HCO-PID controller for ventilator model.

For the system in (17), a PID feedback controller is optimized with the HCO algorithm. The convergence performance of the HCO algorithm to design an optimal PID controller compared with some existing algorithms^27,76^ is presented in Fig. 8a. The performance of the HOC optimized PID controller for the ventilator systems is compared with some existing results as presented in Table 10 and graphically shown in Fig. 8b. It is observed that the response of the ventilator in terms of rise time and settling time with the HCO-PID controller is better than the performance of existing results^27,76^.

**Table 10:** Performance comparison of HCO-PID for respiratory ventilator system.

Method	Controller parameter			Controller performance		
	${{K}_{p}}$	${{K}_{i}}$	${{K}_{d}}$	${{M}_{p}}(\times {{10}^{-4}})$	${{t}_{p}}(s)$	${{t}_{s}}(s)$
Fmincon-PS based PID^76^	1.225	131.95	0.025	1.12	0.005	0.18
C-CTO-PID^27^	1.65	131.267	0.006	1.02	0.005	0.06
HCO-PID	1.76	271.54	0.0034	1.11	0.001	0.002

## Conclusion

In this paper, a novel nature-inspired metaheuristic optimization algorithm named Human Conception Optimizer (HCO) is developed to solve real-world optimization problems. The proposed algorithm is simple to understand and effective. It is based on a natural process that exists because of the evolution of human beings. The ability of the proposed algorithm has been tested with classical CEC-2005 and CEC-2020 benchmark functions (BMFs). A comparative analysis of the HCO algorithm with some existing results has also been performed for both sets of benchmark functions. The simulation results showed the superiority of the proposed algorithm. As observed in Table 3, for most of the CEC-2005 BMFs, the proposed algorithm performed better than existing methods. Moreover, the statistical significance of the HCO algorithm is observed in Table 6 for 30D CEC2020 BMFs. For most complex higher-dimensional test functions, the proposed algorithm performed efficiently. It can be stated that the HCO algorithm can be applied to find solutions for different complex optimization problems as tested with CEC-2005 BMFs, 30D CEC2020 BMFs, and 500D CEC2020 BMFs. For the validation of the proposed algorithm for real world problems, an optimal overcurrent relay coordination problem in a complex distribution network and an optimal PID controller design for an artificial human ventilator system have been examined and compared with existing results. For a complex 14 over-current relay based IEEE 8 bus power distribution system, the proposed method optimized the total relay operating time with optimal coordination among all primary and secondary relays. The total optimal relay operating time achieved by the HCO algorithm for the chosen system is 1.96 seconds, as presented in the Table 9 where it is 8.56 seconds using BBO-LP^69^, 8.69 seconds using BIP^70^, 5.86 seconds using HWOA^71^ and 5.95 seconds using MWCA^73^. A significant improvement in total relay operational time is observed by the proposed algorithm. The initial random population of PS (plug setting) and TDS (Time Dial Setting) for 14 relays in the IEEE 8 bus system is efficiently selected during the generation stage of a healthy population as proposed in the algorithm (Fig. 3). With the proposed algorithm, a gain of 50% to 60% in total relay operating times is observed comparing with some existing results for the same system as presented in Table 9. Thus, the practical novelty of the proposed algorithm is found in this real system. For the other engineering problem of designing an optimal PID controller for a mechanical ventilator model. The convergence performance to find the optimal solution for the ventilator model is better in terms of response time and settling time within acceptable steady state error than existing methods, as found in Fig. 8a. It takes fewer iterations compared with the CTO algorithm to find an optimal solution. Although the PSO algorithm requires fewer iterations than the HCO, the minimum fitness value is achieved by the proposed algorithm compared with both the CTO and PSO. The transient response of the system is also significantly improved by the HCO algorithm, as observed in Fig. 8b. This clearly indicates that the proposed algorithm can perform better than some existing algorithms for handling real-world problems.

Moreover, the practical applications of the proposed algorithm have some limitations with the size of the real-world complex optimization problems, which is clearly observed from the simulation results. For the classical benchmark functions of CEC 2005, the HCO algorithm smoothly converges for most BMFs, as shown in Fig. 4. With an increase in dimension and complexity in BMFs such as 30 and 500 dimensional CEC2020, the HCO faces local stuck problems several times during the simulation and it takes more than 50 iterations to overcome the local trapping problem as observed in Figs. 5c,d, and 6d. In real world applications, the same problem is observed during the simulation. As observed in Fig. 7, with the HCO algorithm, the total relay operational time is found much better than existing result due to the selection of initial searching variables (TDS and PS) efficiently with compromising an initial local trapping problem up-to 10 iterations. Thus, for such a complex optimization problems, there is a limitation to apply the HCO algorithm. As the concept of the natural conception process is directly utilised to model the algorithm, the performance of the HCO algorithm can further be improved by adapting to other schemes like multi-level, mutation, crossover, chaotic search concepts, and so on.

## Data Availability

The corresponding author will disclose the datasets utilized and/or processed throughout the current work upon reasonable request.

## References

[1] Feng L, Sun X, Tian X, Diao K (2022). Direct torque control with variable flux for an srm based on hybrid optimization algorithm. IEEE Trans. Power Electron..

[2] Jin Z, Sun X, Lei G, Guo Y, Zhu J (2021). Sliding mode direct torque control of spmsms based on a hybrid wolf optimization algorithm. IEEE Trans. Ind. Electron..

[3] Jia Y-H, Mei Y, Zhang M (2021). A bilevel ant colony optimization algorithm for capacitated electric vehicle routing problem. IEEE Trans. Cybern..

[4] Das P, Das DK, Dey S (2018). A new class topper optimization algorithm with an application to data clustering. IEEE Trans. Emerg. Top. Comput..

[5] Mirjalili S, Mirjalili SM, Lewis A (2014). Grey wolf optimizer. Adv. Eng. Softw..

[6] Kennedy, J. & Eberhart, R. Particle swarm optimization. In *Proc. ICNN’95-International Conference on Neural Networks*, Vol. 4, 1942–1948 (IEEE, 1995).

[7] Mataifa H, Krishnamurthy S, Kriger C (2022). Volt/var optimization: A survey of classical and heuristic optimization methods. IEEE Access..

[8] Li D (2022). Aging state prediction for supercapacitors based on heuristic kalman filter optimization extreme learning machine. Energy.

[9] Ferro G, Robba M, Haider R, Annaswamy AM (2022). A distributed optimization based architecture for management of interconnected energy hubs. IEEE Trans. Control Netw. Syst..

[10] Sang-To T, Hoang-Le M, Wahab MA, Cuong-Le T (2022). An efficient planet optimization algorithm for solving engineering problems. Sci. Rep..

[11] Du J, Zhang Z, Li M, Guo J, Zhu K (2022). Optimal scheduling of integrated energy system based on improved grey wolf optimization algorithm. Sci. Rep..

[12] Tan KC, Feng L, Jiang M (2021). Evolutionary transfer optimization—A new frontier in evolutionary computation research. IEEE Comput. Intell. Mag..

[13] Jia H, Peng X, Lang C (2021). Remora optimization algorithm. Expert Syst. Appl..

[14] You JB (2021). Machine learning for sperm selection. Nat. Rev. Urol..

[15] Raouf OA, Hezam IM (2017). Sperm motility algorithm: A novel metaheuristic approach for global optimisation. Int. J. Oper. Res..

[16] Shehadeh, H. A., Ahmedy, I. & Idris, M. Y. I. Sperm swarm optimization algorithm for optimizing wireless sensor network challenges. In *Proc. 6th International Conference on Communications and Broadband Networking*, 53–59 (2018).

[17] Shehadeh HA, Idna Idris MY, Ahmedy I (2017). Multi-objective optimization algorithm based on sperm fertilization procedure (mosfp). Symmetry.

[18] Holland JH (1992). Genetic algorithms. Sci. Am..

[19] Rechenberg I, Bergmann HW (1989). Evolution strategy: Nature’s way of optimization. Optimization: Methods and Applications, Possibilities and Limitations.

[20] Koza JR, Poli R, Burke EK, Kendall G (2005). Genetic programming. Search Methodologies.

[21] Van Laarhoven PJ, Aarts EH, Aarts E, van Laarhoven PJ (1987). Simulated annealing. Simulated Annealing: Theory and Applications.

[22] Rashedi E, Nezamabadi-Pour H, Saryazdi S (2009). Gsa: A gravitational search algorithm. Inf. Sci..

[23] Erol OK, Eksin I (2006). A new optimization method: Big bang-big crunch. Adv. Eng. Softw..

[24] Azizi M (2021). Atomic orbital search: A novel metaheuristic algorithm. Appl. Math. Model..

[25] Kaveh A, Talatahari S (2010). A novel heuristic optimization method: Charged system search. Acta Mech..

[26] Dorigo M, Birattari M, Stutzle T (2006). Ant colony optimization. IEEE Comput. Intell. Mag..

[27] Acharya D, Das DK (2020). Swarm optimization approach to design pid controller for artificially ventilated human respiratory system. Comput. Methods Progr. Biomed..

[28] Mirjalili S, Lewis A (2016). The whale optimization algorithm. Adv. Eng. Softw..

[29] Shadravan S, Naji HR, Bardsiri VK (2019). The sailfish optimizer: A novel nature-inspired metaheuristic algorithm for solving constrained engineering optimization problems. Eng. Appl. Artif. Intell..

[30] Srivastava A, Das DK (2022). A bottlenose dolphin optimizer: An application to solve dynamic emission economic dispatch problem in the microgrid. Knowl.-Based Syst..

[31] Rao RV, Savsani VJ, Vakharia D (2011). Teaching-learning-based optimization: A novel method for constrained mechanical design optimization problems. Comput. Aided Des..

[32] He S, Wu QH, Saunders JR (2009). Group search optimizer: An optimization algorithm inspired by animal searching behavior. IEEE Trans. Evol. Comput..

[33] Atashpaz-Gargari, E. & Lucas, C. Imperialist competitive algorithm: An algorithm for optimization inspired by imperialistic competition. In *2007 IEEE Congress on Evolutionary Computation*, 4661–4667 (IEEE, 2007).

[34] Srivastava A, Das DK (2022). Criminal search optimization algorithm: A population-based meta-heuristic optimization technique to solve real-world optimization problems. Arab. J. Sci. Eng..

[35] Wolpert DH, Macready WG (1997). No free lunch theorems for optimization. IEEE Trans. Evol. Comput..

[36] Geem ZW, Kim JH, Loganathan GV (2001). A new heuristic optimization algorithm: Harmony search. Simulation.

[37] Chu, S.-C., Tsai, P.-W. & Pan, J.-S. Cat swarm optimization. In *Pacific Rim International Conference on Artificial Intelligence*, 854–858 (Springer, 2006).

[38] Mucherino, A. & Seref, O. Monkey search: A novel metaheuristic search for global optimization. In *AIP Conference Proceedings*, Vol. 953, 162–173 (American Institute of Physics, 2007).

[39] Lu, X. & Zhou, Y. A novel global convergence algorithm: Bee collecting pollen algorithm. In *International Conference on Intelligent Computing*, 518–525 (Springer, 2008).

[40] Shiqin, Y., Jianjun, J. & Guangxing, Y. A dolphin partner optimization. In *2009 WRI Global Congress on Intelligent Systems*, Vol. 1, 124–128 (IEEE, 2009).

[41] Tan, Y. & Zhu, Y. Fireworks algorithm for optimization. In *International Conference in Swarm Intelligence*, 355–364 (Springer, 2010).

[42] Gandomi AH, Alavi AH (2012). Krill herd: A new bio-inspired optimization algorithm. Commun. Nonlinear Sci. Numer. Simul..

[43] Yang, X.-S. Flower pollination algorithm for global optimization. In *International Conference on Unconventional Computing and Natural Computation*, 240–249 (Springer, 2012).

[44] Eskandar H, Sadollah A, Bahreininejad A, Hamdi M (2012). Water cycle algorithm—A novel metaheuristic optimization method for solving constrained engineering optimization problems. Comput. Struct..

[45] Sadollah A, Bahreininejad A, Eskandar H, Hamdi M (2013). Mine blast algorithm: A new population based algorithm for solving constrained engineering optimization problems. Appl. Soft Comput..

[46] Ramezani F, Lotfi S (2013). Social-based algorithm (sba). Appl. Soft Comput..

[47] Zheng Y-J (2015). Water wave optimization: A new nature-inspired metaheuristic. Comput. Oper. Res..

[48] Mirjalili S (2015). Moth-flame optimization algorithm: A novel nature-inspired heuristic paradigm. Knowl.-Based Syst..

[49] Kashan AH (2015). A new metaheuristic for optimization: Optics inspired optimization (oio). Comput. Oper. Res..

[50] Mirjalili S (2016). Dragonfly algorithm: A new meta-heuristic optimization technique for solving single-objective, discrete, and multi-objective problems. Neural Comput. Appl..

[51] Jaddi NS, Alvankarian J, Abdullah S (2017). Kidney-inspired algorithm for optimization problems. Commun. Nonlinear Sci. Numer. Simul..

[52] Saremi S, Mirjalili S, Lewis A (2017). Grasshopper optimisation algorithm: Theory and application. Adv. Eng. Softw..

[53] Srivastava A, Das DK (2020). A new kho-kho optimization algorithm: An application to solve combined emission economic dispatch and combined heat and power economic dispatch problem. Eng. Appl. Artif. Intell..

[54] Saggiorato G (2017). Human sperm steer with second harmonics of the flagellar beat. Nat. Commun..

[55] Eisenbach M, Giojalas LC (2006). Sperm guidance in mammals—An unpaved road to the egg. Nat. Rev. Mol. Cell Biol..

[56] Kirkman-Brown JC, Sutton KA, Florman HM (2003). How to attract a sperm. Nat. Cell Biol..

[57] Budrikis Z (2020). Sperm swimming is more complicated than thought. Nat. Rev. Phys..

[58] Gaffney EA, Ishimoto K, Walker BJ (2021). Modelling motility: The mathematics of spermatozoa. Front. Cell Dev. Biol..

[59] Raveshi MR (2021). Curvature in the reproductive tract alters sperm-surface interactions. Nat. Commun..

[60] Suarez SS (2008). Control of hyperactivation in sperm. Hum. Reprod. Update.

[61] Leung ET (2022). Simulating nature in sperm selection for assisted reproduction. Nat. Rev. Urol..

[62] Ravaux B, Garroum N, Perez E, Willaime H, Gourier C (2016). A specific flagellum beating mode for inducing fusion in mammalian fertilization and kinetics of sperm internalization. Sci. Rep..

[63] Zhang Z (2016). Human sperm rheotaxis: A passive physical process. Sci. Rep..

[64] Tian F-B, Wang L (2021). Numerical modeling of sperm swimming. Fluids.

[65] Liu Q-Y, Tang X-Y, Chen D-D, Xu Y-Q, Tian F-B (2020). Hydrodynamic study of sperm swimming near a wall based on the immersed boundary-lattice Boltzmann method. Eng. Appl. Comput. Fluid Mech..

[66] Choudhary A, Paul S, Rühle F, Stark H (2022). How inertial lift affects the dynamics of a microswimmer in Poiseuille flow. Commun. Phys..

[67] Lin S, Ke M, Zhang Y, Yan Z, Wu J (2021). Structure of a mammalian sperm cation channel complex. Nature.

[68] Amraee T (2012). Coordination of directional overcurrent relays using seeker algorithm. IEEE Trans. Power Deliv..

[69] Albasri FA, Alroomi AR, Talaq JH (2015). Optimal coordination of directional overcurrent relays using biogeography-based optimization algorithms. IEEE Trans. Power Deliv..

[70] Corrêa R, Cardoso G, de Araújo OC, Mariotto L (2015). Online coordination of directional overcurrent relays using binary integer programming. Electric Power Syst. Res..

[71] Sarwagya K, Nayak PK, Ranjan S (2020). Optimal coordination of directional overcurrent relays in complex distribution networks using sine cosine algorithm. Electric Power Syst. Res..

[72] Korashy A, Kamel S, Jurado F, Youssef A-R (2019). Hybrid whale optimization algorithm and grey wolf optimizer algorithm for optimal coordination of direction overcurrent relays. Electric Power Compon. Syst..

[73] Korashy A, Kamel S, Youssef A-R, Jurado F (2019). Modified water cycle algorithm for optimal direction overcurrent relays coordination. Appl. Soft Comput..

[74] Kida AA, Rivas AEL, Gallego LA (2020). An improved simulated annealing linear programming hybrid algorithm applied to the optimal coordination of directional overcurrent relays. Electric Power Syst. Res..

[75] Hunnekens B, Kamps S, Van De Wouw N (2018). Variable-gain control for respiratory systems. IEEE Trans. Control Syst. Technol..

[76] Sakthiya Ram S, Kumar C, Ramesh Kumar A, Rajesh T (2022). Hybrid optimization techniques based automatic artificial respiration system for corona patient. Automatika.

